# Simulation on the Comparison of Steady-State Responses Synthesized by Transient Templates Based on Superposition Hypothesis

**DOI:** 10.1155/2015/476050

**Published:** 2015-10-27

**Authors:** Xiao-dan Tan, Xue-fei Yu, Lin Lin, Tao Wang

**Affiliations:** School of Biomedical Engineering, Southern Medical University, Guangzhou, Guangdong 510515, China

## Abstract

The generation of auditory-evoked steady-state responses (SSRs) is associated with the linear superposition of transient auditory-evoked potentials (AEPs) that cannot be directly observed. A straightforward way to justify the superposition hypothesis is the use of synthesized SSRs by a transient AEP under a predefined condition based on the forward process of this hypothesis. However, little is known about the inverse relation between the transient AEP and its synthetic SSR, which makes the interpretation of the latter less convincible because it may not necessarily underlie the true solution. In this study, we chose two pairs of AEPs from the conventional and deconvolution paradigms, which represent the homo-AEPs from a homogenous group and the hetero-AEPs from two heterogeneous groups. Both pairs of AEPs were used as templates to synthesize SSRs at rates of 20–120 Hz. The peak-peak amplitudes and the differences between the paired waves were measured. Although amplitude enhancement occurred at ~40 Hz, comparisons between the available waves demonstrated that the relative differences of the synthetic SSRs could be dramatically larger at other rates. Moreover, two virtually identical SSRs may come from clearly different AEPs. These results suggested inconsistent relationships between the AEPs and their corresponding SSRs over the tested rates.

## 1. Introduction

An auditory-evoked steady-state response (SSR) is an evoked potential in response to a periodically sound stimulus. The sound can be of a short impulse, like a click, or a sustained sound, like an amplitude-modulated tone [1]. The auditory SSR is a periodical signal that reflects electrophysiological activity in the auditory nerve system following the driven stimulation [1]. Given that the SSR reflects the response to a regularly changing stimulus, it differs conceptually with the transient auditory-evoked potential (AEP) in which an AEP is supposed to characterize electrophysiological activity in response to a change in the stimulus, for example, its onset or offset [2].

The specific SSR analyzed in this study consists of the periodical superposition of early auditory brainstem response (ABR) and a subsequent middle-latency response (MLR) lasting less than 70 ms. Their featured components, such as wave-V, Na, Pa, Nb, and Pb, are normally characterized by their amplitudes and latencies. According to superposition theory, these components will overlap as the stimulus rate increases and lead to a periodic response or SSR [2, 3]. The amplitude of the resultant SSR can be attenuated or enhanced in agreement with the phase relationship of the waves dependent on the stimulus rate. Therefore, the SSR can be completely predicted at any rate if the underlying transient AEPs are available.

The amplitude of auditory SSRs varies remarkably with respect to the change in stimulus rate and reaches the maximum at approximately 40 Hz [1, 3, 4]. This phenomenon has been largely ascribed to the in-phase summation or overlap of the featured components at that rate [3, 5]. However, this superposition hypothesis is frequently challenged by experiments that found inconsistencies from the predicted SSRs [4, 6]. For example, Azzena et al. [4] reported that the SSRs predicated by conventional ABRs/MLRs at a low stimulus rate are evidently incongruous. The animal model with intracranial recordings exhibited that the synthetic SSRs by AEPs at 3.33 Hz not only overestimated the recorded SSRs, but also showed different variations with increasing stimulus rate [7].

The disagreement of AEP superposition is probably related to the variation in AEP with the stimulus rate [8–10]. Therefore, estimating the individual transient AEP from the overlapped SSRs becomes demanding. As the superposition can be modeled by a convolution process between the underlying AEP and the impulse sequence presenting the onset of the stimulus sound, a number of deconvolution methods have been developed so far to unwrap the SSRs [11–16]. Among these methods, Özdamar et al. [9, 12] proposed a continuous loop averaging deconvolution (CLAD) method and reported the appearance of Pb resonance at 40 Hz. Using transient responses at a high stimulus repetition rate, the SSR prediction can be greatly improved [5, 17–20].

However, other studies have also been reported to challenge the convolution model for the SSR or frequency following response (FFR) [21–23] using specifically designed experiments. For example, Bidelman [23] recently reported the inconsistency in predicting the brainstem FFR elicited by a click at high rates of 100–500 Hz and claimed the functional distinction between the responses of the brainstem FFR and conventional ABR elicited by a click at a stimulus rate of 20 Hz. In addition, using the neuromagnetic response and the stimulation of a two-tone complex modulated by sweeping frequency envelope, Miyazaki et al. [24] associated the perceptual qualities with the critical stimulus rates. They suggested that the evolvement of the transient to steady-state responses has an important perceptual implication of speech. Thus, multiple cortical processes are needed to deal with acoustic events at different time scales. Indeed, experiments conducted by the functional magnetic resonance imaging showed separate auditory cortical representations responding to different stimulus rates [25, 26]. Furthermore, studies from the microscopic level of neuronal activity suggested different mechanisms underlying neural activities responding to the attributes of stimulation, which showed a strong nonlinear phenomenon for the auditory neural system [27].

These findings indicate that invariant transient responses to the isolated stimulus event cannot explain the SSR characteristics at various rates or conditions. The establishment of SSR involves the transition of template variations corresponding to different stimulation paradigms. A number of studies managed to estimate transient AEPs that accommodate conditions used in SSR recordings to make the predicted SSR convincible (e.g., [5, 6]). The inconsistent prediction was claimed to be a failure of the superposition model. Although dramatic variation of the SSRs with stimulus rates was reported, it is still unclear about the rate-effect on the relation between SSRs and their transient templates. Since it is impossible to obtain the true transient response, a simulation of the forward process will be beneficial in understanding the suitability of superposition model. This study aims at investigating how the morphological difference between transient AEPs affects the corresponding synthesized SSRs when the prediction method is used to compare SSRs. We attempted to determine how large the discrepancy is between the difference of SSRs and that of source transient AEPs. We deliberately selected two pairs of AEPs as templates to synthesize SSRs for comparison. The synthesized SSRs and their paired difference waves were compared to exemplify the characteristics of the waves with respect to the stimulus rate.

## 2. Method

### 2.1. Linear Superposition Model

**Figure 1:**
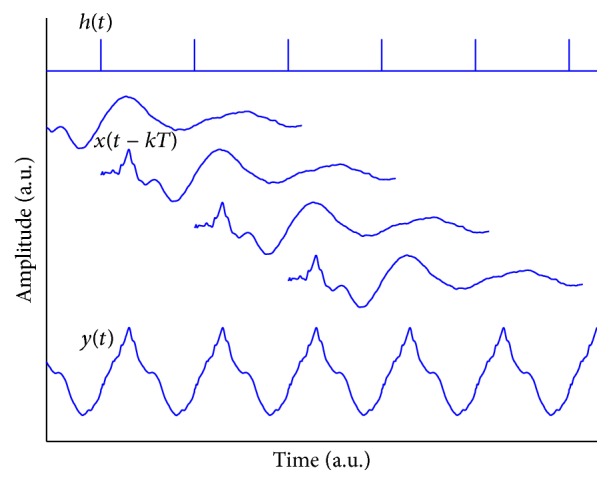
Theoretical diagram illustrating the generation of an SSR under superposition hypothesis. The stimulus sequence *h*(*t*) consists of a series of impulses (digital “1”) spaced at *T*, indicating the onset of a stimulus. The hypothetical respones *x*(*t* − *kT*), *k* = 1,2,…, with different lag in response to the stimuli are superposed to generate the SSR *y*(*t*).

According to the superposition theory, we can model the generation of the SSR by a linear convolution process, in which the SSR, denoted by *y*(*t*), is the convolution result between the underlying transient AEP, denoted by *x*(*t*), and the stimulus sequence, denoted by *h*(*t*) [15, 28]; that is,

(1) $$\begin{array}{c} y\left(\;t\;\right)=x\left(\;t\;\right)⊗h\left(\;t\;\right)+n\left(\;t\;\right), \end{array}$$

where ⊗ denotes the circular convolution operator and *n*(*t*) represents the additive noise. The stimulus-sequence *h*(*t*) is a binary {1,0} train representing the onset of a stimulus with “1” and “0” if otherwise. The interval between “1” represents the inter-stimulus-interval (ISI). If all ISIs are constant in a sequence, a periodical SSR corresponding to that rate will be generated (Figure 1). The model can explain the amplitude enhancement of the SSR at a stimulus rate, particularly at 40 Hz [29], because the interval between the major ABR-V and MLR-Pa is approximately 25 ms. However, using the transient AEP obtained at conventional low rates does not do justice to the validation of the superposition hypothesis, because the influence of the stimulus rate, for instance, the adaption effects on AEPs, fails to be considered [4, 22].

### 2.2. Introduction to CLAD and MSAD Paradigms

Unfortunately, no mathematical solution is available for the source AEP *x*(*t*) under constant ISI conditions [12, 13], unless ISI is adequately large without overlapping. If the SSR is generated by the AEP superposition as formulated in (1), a number of deconvolution methods are available to derive the transient AEPs by the specific sequence design [11–16]. Among these methods, we focused on the following two closely related ones using different sequencing strategies. The CLAD method uses jittered sequences to make the solution of (1) possible. This method can be implemented in the frequency domain via Fourier transform on each of the terms (1):

(2) $$\begin{array}{c} Y\left(\;f\;\right)=H\left(\;f\;\right)X\left(\;f\;\right)+N\left(\;f\;\right). \end{array}$$

The CLAD method can estimate the transient AEP *x*(*t*) at any stimulus rate provided that the stimulus sequence and signal-to-noise ratio are viable. This estimation ignores the jitter effect and yields a kind of averaged response to all stimuli. By contrast, another method, the multirate steady-state averaging deconvolution (MSAD), employs cardinal SSRs at different rates and constitutes a linear transform matrix **H** based on the ISIs at corresponding rates; thus, the convolution model is rewritten by an equivalent linear transform as [15]

(3) $$\begin{array}{c} y=Hx+n. \end{array}$$

The two deconvolution methods are methodologically equivalent. However, the MSAD method uses a different jitter arrangement for the stimulus sequence. A block/session-based ISI variation/jitter for the MSAD is adopted, instead of a real-time jitter in the stimulus sequence like in the CLAD, to make the solution of (3) possible [15].

### 2.3. Transient AEP Template Generation and Synthetic Hypothesis

In the current study on the comparison of AEPs for the deconvolution methods, pilot data were obtained from 20 participants (22 to 26 years, 5 females) in the CLAD and MSAD paradigms at a mean stimulus rate of 40 Hz. By contrast, conventional AEPs of 5 Hz were also obtained. The experiment was in accordance with an IRB-approved protocol. The stimulus sequence of the CLAD paradigm was obtained in the literature [9] that contains eight clicks with different ISIs from 16 to 36.8 ms in a stimulus sweep. Eight rates from 27 to 62.5 Hz of a single cycle of SSRs were obtained for the MSAD paradigm. The recording setting and data processing procedure can be found in detail as described in [9, 12, 30].

If the linear superposition hypothesis is valid, the models of (2) and (3) should be equivalent to the resembling solutions. However, morphological differences among AEPs are clearly identified for different paradigms. The physiological cause for this phenomenon may be associated with different adaptations of neuronal systems, because the jitter distribution has been found to affect the neural response by fast and slow mechanisms of adaptation [31]. In the present study, we intentionally selected two pairs of AEP from these data as templates to investigate their contributions to the synthetic SSRs, regardless of the physiological mechanism for these paradigms.

**Figure 2:**
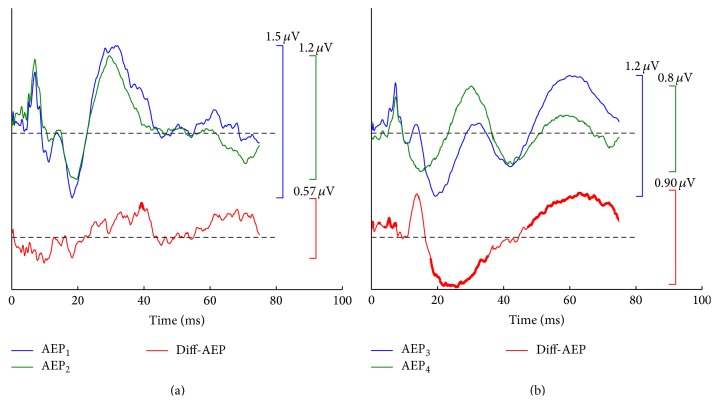
Two pairs of transient AEP templates and corresponding peak-peak amplitudes. (a) AEP_1_ and AEP_2_ for homocondition and their difference waves. (b) AEP_3_, derived from the CLAD, and AEP_4_, derived from the MSAD, for hetero-AEPs and their difference waves. The heightened portions on the difference waves indicate the statistical significance along the whole time course over all individuals. All the labeled peak-peak amplitudes were used as references in the SSR comparisons mentioned in Section 3.

For the first AEP pair, we arbitrarily dichotomized 20 individual data sets of the conventional paradigm to yield two averaged AEPs, that is, AEP_1_ and AEP_2_, in Figure 2(a). Therefore, this AEP pair, referred to as* homo-AEPs*, was sampled from the same recording condition with resembling morphology (Figure 2(a)). Their difference wave exhibited a low amplitude and a random pattern (second row in Figure 2(a)). We statistically analyzed the significant difference at every sampling point along the whole time course and then measured the morphological difference in terms of a* significant percentage*, which is defined as the ratio of the total number of sampling points with significant difference over all points of the whole wave. Through this measurement, we found almost no significant difference along the time course, except for a minor piece highlighted in bold (Figure 2(a), two-tailed* t*-test, *p* < 0.05). The significant percentage was only 2% over the entire time course.

The second AEP pair was separately selected as a pair of* hetero-AEPs* from the averages in the CLAD and MSAD paradigms, that is, AEP_3_ from the CLAD and AEP_4_ from the MSAD (Figure 2(b)), which exhibited a large morphological difference (second row, Figure 2(b)). The sampling points with significant difference (highlighted in bold) covered as large as 64% (paired two-tailed* t*-test, *p* < 0.05). The peak-peak amplitudes for these AEP templates and corresponding difference waves served as a reference when dealing with the SSRs in the following comparisons. The SSRs at various stimulus rates were then synthesized by the selected AEPs for homo- and heteroconditions. The differences among the synthetic SSRs were also analyzed.

## 3. Results

### 3.1. Comparisons of the SSR Amplitudes

**Figure 3:**
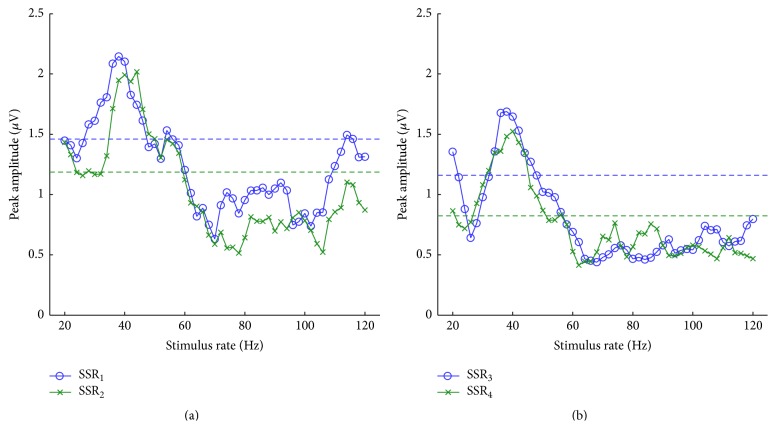
Amplitudes of SSRs as a function of stimulus rates. (a) Amplitudes of SSR_1_ and SSR_2_ for homocondition. (b) Amplitudes of SSR_3_ and SSR_4_ for heterocondition. The blue horizontal line represents the peak-peak amplitude of AEP_1_ in (a) and that of AEP_3_ in (b). The green line represents the peak-peak amplitude of AEP_2_ in (a) and that of AEP_4_ in (b).

Based on the two pairs of AEP templates, we then synthesized the SSRs at stimulus rates of 20–120 Hz with an increment of 2 Hz. In relation to the rates, the peak-peak amplitudes of SSRs are shown in Figure 3. All the SSR amplitudes demonstrated a similar profile: a striking peak at ~40 Hz and fluctuations over the other rates. Although amplitude enhancements were observed at certain rates, such as at ~55 and ~115 Hz in Figure 3(a), the corresponding peak-peak amplitudes were similar to the corresponding reference levels (horizontal lines), indicating that the SSRs clearly surpassed the AEPs only at a range of rates around 40 Hz.

The amplitude of the homo-SSR pair (Figure 3(a)) exhibited approaching (e.g., in 50–70 Hz) and parted (e.g., in 20–35 and 110–120 Hz) inclinations. By contrast, hetero-SSRs exhibited a relatively close amplitude for most rates (Figure 3(b)), even though the original AEPs differed greatly (see Figure 2(b)). These results suggested that synthetic SSRs may exhibit an approaching or parted amplitude at different rates and under different conditions.

### 3.2. Comparisons of the SSR Differences

**Figure 4:**
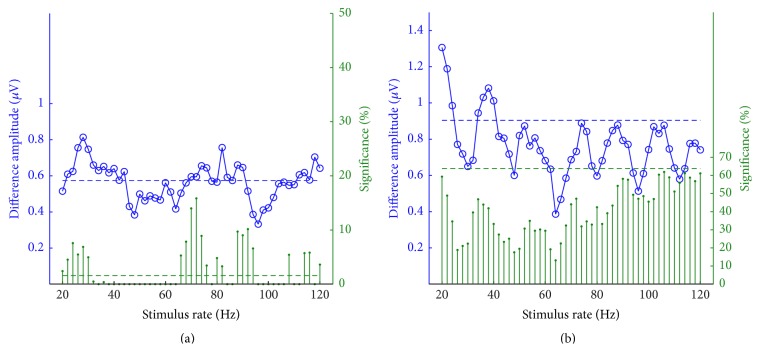
Absolute differences of the SSRs (blue “-○” traces) and significant percentages (green vertical lines) over the stimulus rates under (a) homo- and (b) heteroconditions. The blue horizontal lines indicate the peak-peak amplitude of original AEP differences in Figure 2, and the green ones indicate the significant percentages of original AEP difference waves.

In line with the difference waves of AEPs, we could produce the SSR-difference (diff-SSR) waves between SSRs in both conditions. The peak-peak amplitudes of the diff-SSRs over all rates are shown in Figure 4 (blue “-○” traces), which exhibited relatively even fluctuation over all the available rates without dominating extremes. The diff-SSR amplitudes in the homocondition basically fluctuated across the reference (blue dotted line in Figure 4(a)), whereas the amplitudes of the hetero-diff-SSRs were all lower than their reference (blue dotted line in Figure 4(b)). In addition, a regular oscillation pattern appeared in the amplitude trace for heterocondition.

For diff-SSR waves, the significant percentage as defined in Section 2.3 was also calculated as an index reflecting the largeness of the statistical difference of an SSR pair. The percentages of significant differences were presented in the same coordinates with diff-SSRs (green vertical lines in Figure 4). These values largely fluctuated over the rates for both conditions. Specifically, the percentages of 0 to more than 15% for homocondition, and the percentages of ~20% to ~60% for heterocondition, all demonstrated an unpredictable phenomenon over the testing rates. However, they presented a minor positive correlation with the diff-SSRs in both conditions (*r* = 0.31 for homoconditions and *r* = 0.27 for heteroconditions, *p* < 0.05), suggesting that the larger the SSR difference is, the more likely the differences were significant. Although the absolute peak-peak amplitudes of SSRs under the heterocondition were generally smaller compared with that under the homocondition (see Figure 3), their differences were still generally larger because of the large differences between the original AEP_3_ and AEP_4_.

**Figure 5:**
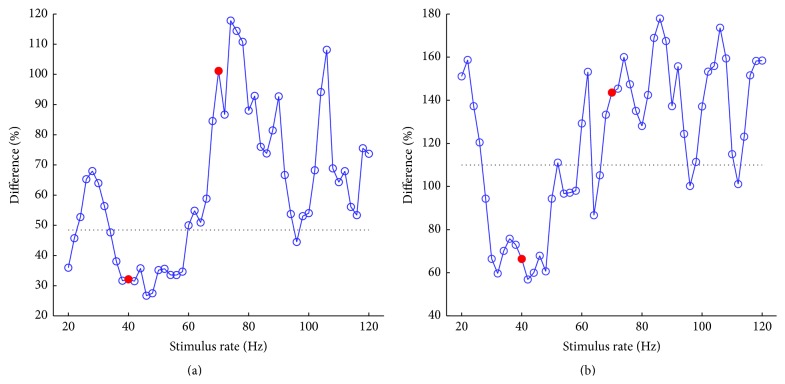
Relative differences of SSRs as a function of the stimulus rates for (a) homo- and (b) heteroconditions. The horizontal dotted lines indicate the relative differences of original AEPs. Two representative rates, that is, 40 and 70 Hz (filled circles in red), are selected for further analysis in Section 3.4.

Given that the peak-peak amplitudes for SSRs and diff-SSRs behaved differently over the stimulus rates, the relative difference in terms of the ratio between the amplitudes of diff-SSRs and SSRs (defined as the minimum one of a pair of SSRs) is presented in Figure 5. Basically, these values converged to the minimum at ~40 Hz with a few samples less than the original AEP references (horizontal dotted lines in Figure 5). Other samples beyond ~60 Hz fluctuated dramatically with a number of samples as large as more than 100% even for the homocondition (Figure 5(a)), which indicated that the diff-SSRs were even larger than the SSRs. The relative difference for hetero-SSRs shared a similar pattern to even larger ratios (Figure 5(b)). As such, one must be cautious in interpreting the similarity relation between SSRs and the original AEPs. These simulation results implied that the identity of the underlying AEPs could hardly be predicted stably from the resemblance of SSRs at certain rates, except for some rates close to the enhanced range (e.g., ~40 Hz) in which the least relative differences occurred.

**Figure 6:**
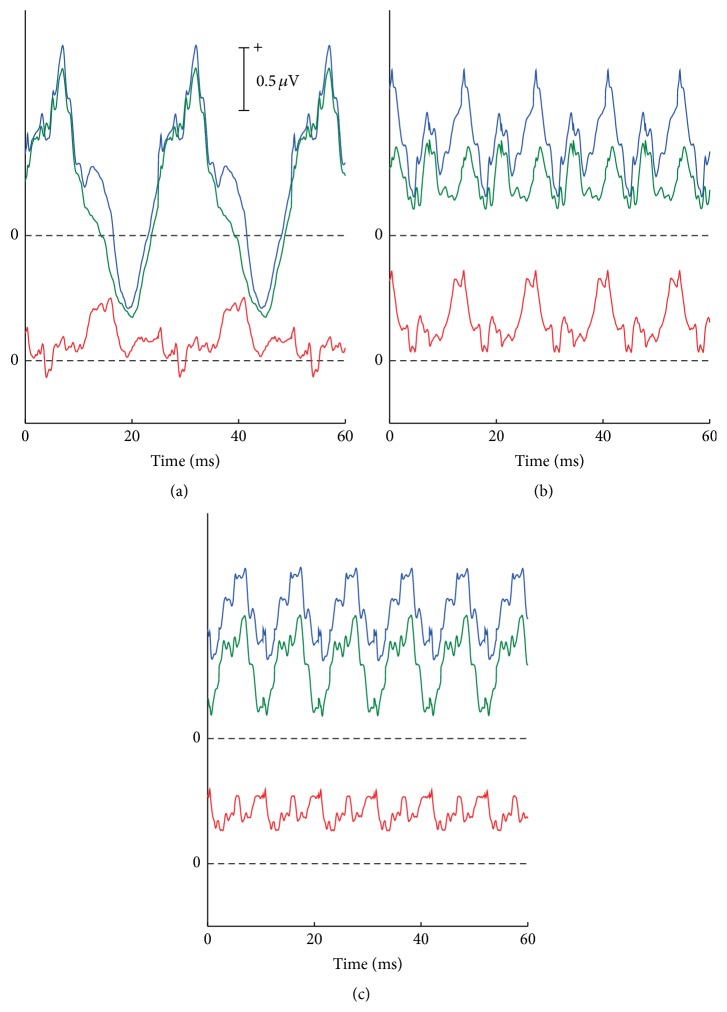
Representative waves of synthetic SSR_1_ (blue wave) and SSR_2_ (green wave) and corresponding diff-SSRs (bottom rows) at three representative stimulus rates (40, 74, and 96 Hz).

Based on these results, the SSRs and the diff-SSRs exhibited diverse features at different stimulus rates. Three representative stimulus rates (40, 74, and 96 Hz) were selected to compare the SSR waves with the diff-SSRs for the homocondition (Figure 6). The largest SSRs at 40 Hz (Figure 6(a)) were visually close in morphology, and a relatively moderate diff-SSR showed that the most remarkable difference did not occur on the peaks or troughs, which were the pivotal portions of SSRs in applications. The waves at 74 Hz (Figure 6(b)) demonstrated a relatively larger diff-SSR for two moderate SSRs, and the largest difference appeared at the pivotal peaks and troughs. Figure 4(a) illustrates that the percentage of significant difference was also relatively large (~10%). The waves at 96 Hz (Figure 6(c)) demonstrated that two synthetic SSRs resembled each other very well and were relatively low in amplitude. This case indicated the possibility that the difference of synthetic SSRs could be virtually neglected and even synthesized from the clearly different AEPs.

### 3.3. Simulation of Identical SSRs from Different AEPs

**Figure 7:**
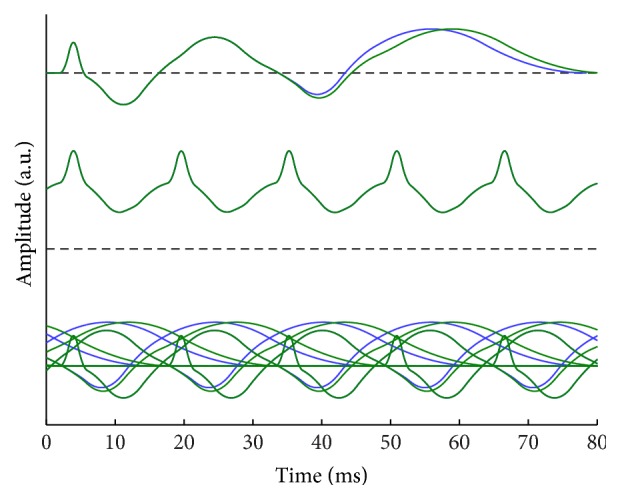
Schematic illustration of a case of virtually identical SSRs synthesized by two clearly different transient AEPs at a rate of 64 Hz.

To illustrate the possibility for virtually identical SSRs to be synthesized by clearly different AEPs, we deliberately designed two artificial AEP templates (first row in Figure 7). The two templates were generated by a superposition of a number of spline functions to simulate typical AEP waves. The shapes of both templates were identical, except for a clearly different latency on the last positive component (corresponding to Pb in a typical AEP wave). When synthesizing the SSRs at a rate of 64 Hz (second row), they were virtually identical by visual inspection. The SSRs were all in positive polarity, with the waves all above the baseline. We checked the superposing process of the AEP templates for the coincidence's mechanism (third row). The difference appeared all over the time course because of phase shifts. A clear phase difference resulted in the same summation waves. Specifically, the summation of Pb waves was flat at this particular phase lag, thereby eliminating the difference caused by phase shifts. This result implied that the SSRs may be insensitive to latency shifts in some cases.

### 3.4. Comparison of SSRs in the Frequency Domain

**Figure 8:**
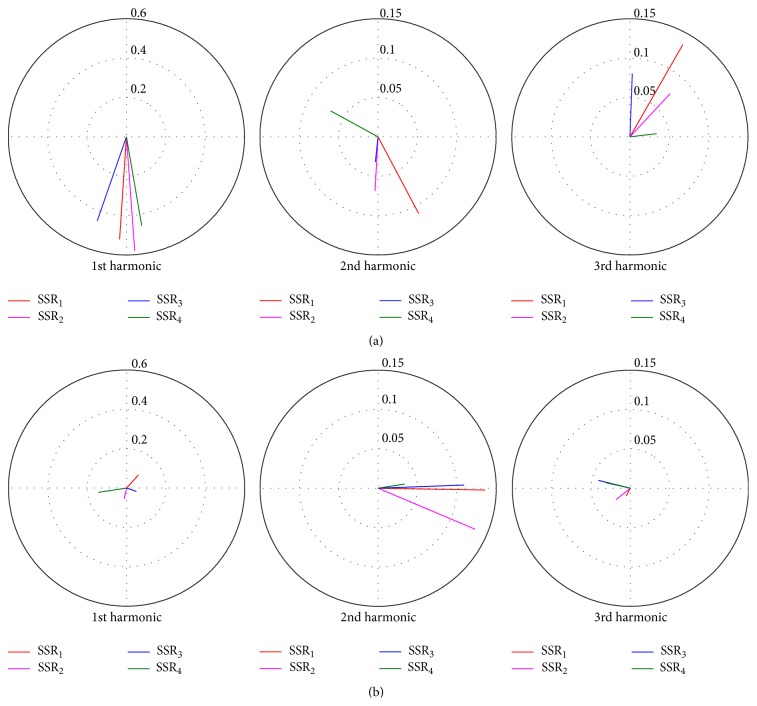
Phasor diagram of SSRs from SSR_1_ to SSR_4_ for the first three harmonics in polar coordinates. The SSRs at 40 (a) and 70 Hz (b) are selected for comparison.

The SSRs are generally characterized in the frequency domain, because periodical signals can be adequately approached by a summation of a number of harmonics. Therefore, we deliberately compared the synthetic SSRs under homo- and heteroconditions. We selected two stimulus rates of 40 and 70 Hz to represent the lowest and highest extreme cases in terms of relative differences in SSR, respectively (see the filled circles in Figure 5). The first three harmonics were used to represent the frequency characteristics of an SSR. A two-tuple phasor that represents the amplitude and the phase of a harmonic constituent completely represents the sinusoid component in the time domain. In this way, Figure 8 illustrates the constituent phasors for the first three harmonics in the polar coordinates.

In the case of 40 Hz SSRs (Figure 8(a)), the first harmonics of 40 Hz that accounted for the largest portion of SSRs showed generally comparable amplitudes and phases. Given that SSR_1_ and SSR_2_ were from the same recording paradigm, they were relatively close for all harmonics. The 40 Hz phasors for SSR_3_ and SSR_4_ were more apart in phase. For the second harmonics (80 Hz phasors), SSR_4_ was roughly opposed and reversed to other phasors. Large differences also occurred in the third harmonics (120 Hz phasors). This result for 40 Hz SSRs showed that the harmonic representation characterized the main frequency properties, consistent with the underlying transient AEPs from the two conditions. The largest first harmonic amplitude indicated that the 40 Hz frequency composition could capture the SSR wave as well.

In the case of 70 Hz SSRs (Figure 8(b)), the amplitudes of the first harmonics were smaller than the amplitude of the second harmonics. Meanwhile, these phasors were completely out of phase. The second harmonics played a dominant role with relatively consistent phasor directions.

Using the phasor diagram method, we compared the main structure of SSRs and found a major discrepancy in the frequency constituent. The results for the two representative rates indicated that the fundamental harmonic failed to capture the main temporal structure of the SSRs at some stimulus rates, and the distinction for SSRs also became vague in comparison with the resonance rates (e.g., 40 Hz).

## 4. Discussion

The relationship between transient AEPs and SSRs is a major concern in speculating the generation of SSRs. No direct evidence is available to support or reject the superposition hypothesis because of the complexity of the underlying neural connection and activation. Thus, an efficient approach to address this relationship is to compare the SSRs between a true experiment and a referential synthesis from a conjectured AEP. If these two waves matched well, a positive conclusion will be accepted. This notion is based on the proposition that approaching AEPs will definitely produce approaching SSRs. However, the results of this study presented negative evidence under certain conditions based on the simulation experiment. The SSR prediction from available transient AEP templates may vary dramatically at some stimulus rate other than the rates close to the most enhanced amplitude condition (i.e., 40 Hz in this experiment). This conclusion may partly explain some discrepancies reported between the predicted and recorded SSRs. For example, Lütkenhöner and Patterson [22] reported that synthetic SSR could completely predict the SSRs at 40 Hz but failed at 60 Hz. The amplitude of 60 Hz [22] was largely attenuated, indicating that a large relative difference might occur, as in the case of Figure 6.

The templates used in this experiment were AEPs from a conventional low rate (~5 Hz) and two deconvolved high rates (~40 Hz). Their morphological differences are likely associated with the adaptation of the neuronal system [8–10, 31]. This mechanism may account for the inconsistency of the prediction with the templates at other stimulus rates or even with another stimulus sequencing [21, 22]. For example, Valderrama et al. [31] reported that the fast and slow mechanisms of adaptation may account for the AEPs' difference in response to the jitter distribution and stimuli sequencing. In a visual event-related potential study, Capilla et al. [19] reported a transient template estimated from the same stimulus rate, as the SSR can adequately predict the SSR from 7.7 Hz to 20 Hz. As such, the nonlinearity of the auditory system does not contradict the superposition of the SSR, if the estimated templates can take these factors into account. The characteristic of transient responses depending on stimulation paradigms is likely to reflect the nonlinear or adaptation mechanisms of neuronal activity. Thus, further studies with the help of specifically designed experiments are necessary.

In theory, the transient AEP is adequate to offer all the information present in the SSRs because an SSR is a filtered version of an AEP in which the filter is an impulse train in the time domain [32]. This definition means that information loss is inevitable for SSRs with respect to the underlying AEPs. As shown in Figure 7, the ill-posedness of the superposition model suggested that even the matched prediction did not necessarily mean it was the sole solution for the SSRs. Consequently, the prediction results should be interpreted with caution.

The frequency analysis demonstrated that the major energy of SSRs at different stimulation rates may occur at different harmonics [33]. For example, the synthetic 40 Hz SSR was adequately approached by the summation of 40, 80, and 120 Hz harmonics. The maximum energy occurred at 40 Hz, which was ascribed to the maximum amplitude enhancement at this rate. By contrast, the maximum energy for 70 Hz SSR existed at the second harmonic, because the latency difference of two adjacent positive/negative peaks in the AEP was about twice the ISI at 70 Hz. The depression of the fundamental harmonic with respect to others was also reported by Miyazaki et al. [24]. The frequency analysis selected at two representative rates demonstrated that the contribution of harmonics to an SSR was also rate-dependent.

The AEP templates were from averaged recordings of three paradigms. The number of individual AEPs was 10 for homogroup and 20 for heterogroup. Both the recording paradigm and the number of individual AEPs would affect the signal-to-noise ratio for the averaged AEP templates. Thus, the comparison of the averaged signal template themselves would be of little value or importance, whereas the variation of individual waves can be accommodated in the testing of the significant difference. AEPs from the same group (homo-AEPs) may result in a more significant difference when used to synthesize SSRs; even the amplitude of diff-SSRs was not greatly increased (for instance, the case at ~70 Hz in Figure 4(a)). Unlike the rate effect on SSR amplitudes, which showed SSR enhancement or attenuation at some specific rates, no clear rate effect on diff-SSRs was found for both homo- and heteroconditions.

The magnitude of the SSR fluctuates with the stimulus rate: that is, the enhancement and attenuation appear to be alternative, which cannot be explained exclusively by neuronal adaptation mechanism [1, 7]. Obtaining templates for all available rates is current unavailable in this study; nevertheless, the rate effect on the SSR [1] is in general coincident with the superposing process shown in Figure 3, implying the existence of the superposition mechanism in generating SSRs.

In clinical settings, the SSR recording at ~40 Hz is adopted to benefit the higher signal-to-noise ratio compared with AEPs for hearing assessment [34, 35]. Other stimulus rates, such as ~90 Hz, were also reported to be enhanced in amplitude [1]. Nevertheless, SSRs at ~40 Hz actually surpassed the amplitude of corresponding AEPs (see Figure 3).

The SSR results of this study were derived from a few templates. We did not intend to generalize the findings. Some exhibited relationships may only be valid within the case of the templates. Nevertheless, these templates were representative samples obtained from both classic and burgeoning paradigms with high stimulus rates. The implication of a variable correlation between SSRs and AEPs is still enlightening and beneficial for future investigations.

In summary, this study provides insight into the relationship between the transient AEP and synthetic SSR at different stimulus rates under the superposition hypothesis. By simulating SSRs over a range of stimulus rates, we demonstrated three rate effects on the SSR: (1) the superposition can be less evident at some rates when the amplitude attenuation occurs; (2) the ill-posedness at certain rate will make the prediction method less convincible; (3) the fundamental frequency components may not be dominated at certain rate. These results suggest that an inconsistent relationship exists between AEPs and SSRs over these rates. Caution should be taken when dealing with the comparison of SSRs over some stimulus rates using the synthetic method.
